# Literary Notes

**Published:** 1899-05

**Authors:** 


### LITERARY NOTES.

---

THE *Therapeutic Digest* closed with last month's issue on account of the death of the business manager, and the recent and continued illness of the editor.

---

LEA BROTHERS & CO., medical publishers, Philadelphia and New York, announce the early publication of Gerrish's anatomy by American authors. Its editor, Prof. F. H. Gerrish, of Portland, has selected as his fellow contributors leading anatomists throughout the country, restricting their number to accord with the best division of the subject. The list includes Professors Bevan, of Rush Medical College; Keiller, of the University of Texas; McMurrich, of the University of Michigan; Stewart of the University Bellevue College, New York; Woolsey, of Cornell Medical College, and the editor.

The plan of the work is to reserve its space for those portions of anatomical knowledge which are necessary to the intelligent study of physiology, surgery and internal medicine. Pictorially it will be the most lavish work yet offered. The engravings number about 1,000, their size is large enough to make visible every detail, colors have been employed more liberally than ever before, and lastly the labels of the parts have been conspicuously engraved upon them whereby a glance gives not only their names, but also their position, extent and relations, obviating entirely the slow, toilsome and wasteful mental processes necessitated where only reference letters are employed.

---

THE *International Review* of Rhinology, Otology and Laryngology has been enlarged by the addition of a new department, called experimental phonetics. The progress of phonetics in the past has been closely allied with the progress of physiological linguistics. Special physicians by their observations on the organs of the voice in their normal and pathological conditions have already rendered the greatest service, and it has seemed to the editor of the *Review* and also to the director of the laboratory of experimental phonetics of the College of France, that it would be beneficial, both to linguists and special physicians to establish such a department in this journal. The *Review* thus enlarged contains about 80 pages.

---

THE Medical Society of the County of Kings has published its history, which embraces the official program of the Greco-Roman Festival to Asklepios and Esculapius, given under the auspices of the woman's auxiliary to the building committee. It is an elaborately illustrated brochure, handsomely printed and bound, and reflects great credit upon the press committees, Mrs. Havens Brewster Bayles, business chairman, and Mrs. Homer L. Bartlett, literary chairman. We could wish that the Medical Society of the County of Erie would demonstrate a like commendable enterprise and proceed to the erection of a building of its own. What a splendid way it would be to inaugurate the beginning of a new century, and how dignifiedly the medical profession could, with such a building, entertain the physicians who will visit Buffalo during the Pan-American exposition.

---

THE Antikamnia Fetation and Parturition Chart is a useful reference tablet, illustrated in color from drawings made by the late Dr. Louis Crusius, of St. Louis. It begins with the date of fertilisation and carries the development of the fetus on to term. The drawings possess great anatomic accuracy and the accompanying descriptions are all that could be desired. An excellent parturition table is found at the end of the chart. It can be obtained by any accredited physician upon addressing the Antikamnia Chemical Company, 1723 Olive street, St. Louis.

